# Trends in Mortality After the Roll‐Out of Directly Acting Antivirals for Hepatitis C in England Using Linked Surveillance Data

**DOI:** 10.1111/jvh.70210

**Published:** 2026-07-12

**Authors:** David Etoori, Ruth Simmons, Annabel Powell, Monica Desai, Paul Trembling, Caroline Sabin, William Rosenberg

**Affiliations:** ^1^ Centre for Clinical Research, Epidemiology, Modelling and Evaluation (CREME), Institute for Global Health University College London London UK; ^2^ Health Protection Research Unit (HPRU) in Blood‐Borne and Sexually Transmitted Infections at UCL National Institute for Health and Care Research (NIHR) London UK; ^3^ Blood Safety, Hepatitis, Sexually Transmitted Infections and HIV Division UK Health Security Agency London UK; ^4^ Institute for Liver and Digestive Health, Division of Medicine University College London, Royal Free Hospital London UK

**Keywords:** directly acting antivirals, hepatitis C, life expectancy, mortality, sex distribution

## Abstract

Directly acting antivirals (DAAs) revolutionised hepatitis C virus (HCV) treatment, offering higher cure rates. We aimed to quantify life expectancy (LE) differences between treated and untreated individuals diagnosed with HCV and assess the contributions of age and causes of death to these differences. We used routine HCV surveillance data linked with UK mortality records. Individuals contributed person‐time from age 15 or the earliest HCV diagnosis, whichever was later, and were classified as treated six months post‐treatment initiation. The study was divided into two periods: 2015–2018 (Period 1), representing early DAA roll‐out targeting advanced liver disease, and 2019–2022 (Period 2). We used non‐parametric survival analysis methods and mortality decomposition methods to estimate LE differences, and age‐ and cause‐specific contributions to LE differences. A total of 92,920 individuals contributed 322,570 untreated and 200,581 treated person‐years of observation, and 13,294 deaths to the analyses. In Period 1, there was an 8.5‐year LE advantage at age 15 for treated females (95% CI: 5.4–11.5) and 8.9 years for treated males (95% CI: 5.6–12.2). In Period 2, this declined to 6.7 (95% CI: 3.3–10.2) and 5.3 (95% CI: 3.0–7.6), respectively. The narrowing gap was driven by rising deaths from suicide, alcohol, and drug misuse in the treated population and corresponding declines in the untreated population. Treated individuals with HCV had consistently higher LE, though the advantage decreased over time. We hypothesise that the early prioritisation of individuals with cirrhosis resulted in a relatively healthier untreated population (i.e., fewer cirrhotic cases) in the later period.

**Abbreviations**
CLD: Chronic Liver Disease
CoD: Cause of death
DAAs: Directly acting antivirals
D&A: Drug and alcohol services
ESLD: End‐stage liver disease
HCC: Hepatocellular carcinoma
HCV: Hepatitis C
ICD‐10: International Classification of Diseases version 10
LE: Life expectancy
NHS: National Health Service
ODN: Operational delivery network
ONS: Office for National Statistics
PWID: People who inject drugs
SADAL: Suicide, alcohol and drug toxicity, and alcohol‐related liver disease
SSBBV: Sentinel surveillance of blood‐borne virus testing
SVR: Sustained virologic response
UKHSA: The UK Health Security Agency
VCSE: Voluntary, community and social enterprise groups
WHO: The World Health Organisation

## 1 Introduction

The World Health Organisation (WHO) estimates that 50 million people are living with chronic hepatitis C virus (HCV) infection, with approximately one million people newly acquiring HCV infection per year worldwide. The WHO also reported that in 2022, an estimated 242,000 people died from HCV‐related causes, primarily cirrhosis and hepatocellular carcinoma (HCC) [1].

The HCV landscape has undergone a shift following the introduction of directly acting antivirals (DAAs) [2]. Since their roll‐out in England in 2014, these regimens have replaced older, more toxic therapies, offering high cure rates and shorter treatment durations [3]. The primary goal of HCV therapy is sustained virologic response (SVR), which is effectively a cure and is strongly associated with reduction in liver disease progression, a decrease in the incidence of HCC, and a lower risk of all‐cause mortality [4, 5]. Despite these clinical breakthroughs, the population impact on mortality remains complex. Questions remain about DAA long‐term benefits, particularly among people who inject drugs (PWID) who experience high morbidity and mortality, often due to causes not linked to HCV infection [6, 7]. Additionally, those with a long history of infection may already have advanced cirrhosis or comorbidities that may continue to influence their survival trajectories even after virus eradication.

In England, the hepatitis C elimination programme, led by the National Health Service (NHS), was established in 2015 to eliminate HCV as a public health threat by 2030, in line with WHO targets [8]. The programme offers free, accessible testing through community services, an online portal, general practices, and hospitals. Free treatment is available for those who test positive through Operational Delivery Networks (ODNs), which are regional formal NHS structures in which stakeholders work together to optimise healthcare delivery and coordinate care pathways. Between 2015 and 2023, 78.3% of individuals diagnosed with chronic HCV were recorded as having initiated treatment in surveillance data [9].

The programme also works with drug and alcohol services to offer bespoke services in these settings, offers peer support and education through the Hepatitis C Trust charity, and runs a national initiative to find and treat hard‐to‐reach groups, which has included a national reengagement exercise [10, 11] and opt‐out testing in emergency departments [12, 13]. As a result of these efforts, the number of people living with chronic HCV infection fell by 56.7% between 2015 and 2023 to an estimated 55,900 [9]. Much of this reduction in prevalence was due to the introduction of DAAs. There have also been declines in mortality and morbidity associated with end‐stage liver disease (ESLD) and HCC [9].

This paper aims to describe mortality patterns and age‐ and cause‐specific contributions to differences in all‐cause mortality between treated and untreated populations using linked surveillance data. We seek to identify the remaining drivers of mortality in these groups. Eight years of data allow an evaluation of long‐term mortality trends as the DAA era has matured. We report on life expectancy (LE) at age 15 by treatment status in England between 2015 and 2022. LE is a well‐suited metric as it is age‐standardised and values both reductions in deaths and increased longevity (i.e., the age at which death occurs). Our analyses focus on the LE deficit, defined as the shortfall of the LE in untreated individuals compared to those who have received DAA treatment, and we use demographic decomposition techniques to estimate the contribution of changes in liver‐related and other causes of death to the LE deficit.

## 2 Methods

### 2.1 Data and Study Population

Data for this study come from the UKHSA Sentinel Surveillance of Blood‐borne Virus testing (SSBBV), which collects information on hepatitis viruses A to E, HIV, and human T‐lymphotropic virus type 1 (HTLV) tests, regardless of the result. The SSBBV started in 2002 and currently includes 23 laboratories [14, 15]. For our analyses, individuals with an HCV RNA‐positive result in SSBBV with no evidence of referral for treatment (i.e., no record in the HCV treatment registry) were considered untreated.

Treatment information comes from the HCV patient registry and treatment outcome database (NHS registry), which stores records of all individuals referred to ODNs for DAAs.

Disease progression metrics (e.g., fibrosis) are primarily measured for individuals identified as being at increased risk of liver disease, often as part of targeted screening programmes, based on existing clinical conditions, or as part of treatment initiation. As such, those in care have a higher likelihood of recorded tests. Liver fibrosis is assessed by either histological analysis of liver biopsy, liver elastography, or direct (e.g., Enhanced Liver Fibrosis (ELF) test) or indirect (e.g., Fib‐4) non‐invasive blood tests.

Mortality data come from the Office for National Statistics (ONS), where all deaths in England and Wales are registered by age, sex, and underlying causes. All deaths must be registered by law, ensuring near‐complete population coverage.

All data were linked deterministically using five variables (NHS number, first name, last name, date of birth, and sex).

### 2.2 Data Preparation

Individuals successfully treated for HCV before 2015 (DAAs or interferon) were excluded from our analyses. Individuals contributed person‐time to the analyses from their fifteenth birthday or their earliest HCV diagnosis date, whichever was later (i.e., if HCV diagnosis occurred after age 15, then individuals contributed person‐time from their age at diagnosis; if diagnosis occurred before age 15, then individuals contributed person‐time from age 15). Individuals were classified as treated six months after their most recent treatment date (Appendix 1), with this time interval being chosen to account for 8–12 weeks of treatment and the first SVR test, which is measured at least 12 weeks after treatment completion. They continued to contribute to the untreated population until the end of the six‐month period, and any mortality in this period was considered in the untreated group. Treated individuals were assumed to remain HCV RNA negative until the earliest of either the date on which their follow‐up was censored or the date of death. We believe this is reasonable as between 2015 and 2023, the rate of reinfection with hepatitis C was 6.4 per 100 person‐years [9].

ONS cause of death (CoD) data is reported as up to twenty ICD‐10 codes for each death. To categorise causes of death, we used the first three ICD‐10 codes following advice from clinicians. CoD was assigned using the first cause; if this was not specific enough, then the second and third causes were considered. Starting with recognised ICD‐10 categories, causes of death were then amalgamated into broader categories based on their relationship to HCV (e.g., liver‐related causes) and their frequency in the data (e.g., less frequent causes were grouped under ‘Other’). For illustrations, deaths due to suicide, alcohol and recreational drug toxicity and alcohol‐related liver disease are amalgamated as SADAL [16, 17] (Appendix 2).

### 2.3 Statistical Analyses

We present differences in adult LE by sex at birth (male, female), calendar period [2015–2018 (Period 1) and 2019–2022 (Period 2)] and DAA treatment status (treated, untreated) in England between 2015 and 2022. Adult LE is defined as the number of additional years a person of age 15 can expect to live if they are subject to the observed mortality conditions in each period. Estimates of adult LE were computed using continuous‐time survival analysis as the area under the Kaplan–Meier survival curve (referred to as the restricted mean survival time in epidemiologic literature [18]). Confidence intervals for LE differences were computed using bootstrapping with 1000 replications and corrected for bootstrapping bias.

The two calendar periods were selected because 2015–18 represents the early DAA era, when individuals with advanced liver disease were prioritised for treatment, and 2019–2022 represents the later DAA era with more widespread use of DAAs. By categorising calendar years in this way, the two periods included sufficient data to allow us to stratify our analyses and reduced the influence of mortality changes in any single year, allowing us to decompose the total LE difference due to treatment into contributions resulting from mortality differences in five‐year age groups. Using cause‐specific mortality fractions, we estimated the cause contributions to treatment differences in adult LE for each age group. Age group and cause‐specific contributions were calculated using the Arriaga method [19]. In brief, this technique breaks down the overall difference in life expectancy between two populations (in our case treated vs. untreated cohorts) by measuring the direct effect of differing mortality within each age group (i.e., more people surviving in a specific age group), the indirect effect (i.e., those who survive as a result of differing mortality experiencing the mortality rates in older age groups), and an interaction effect (which accounts for the fact that mortality rates are changing across all age groups simultaneously). Using mortality fractions, contributions by each age group are further broken down by causes of death. By doing this, the method provides a precise age and cause breakdown of what is driving observed differences in life expectancy between two groups [20]. Analyses were performed separately for each period.

#### 2.3.1 Sensitivity Analyses

We run sensitivity analyses varying the treatment classification lag (to immediately following treatment initiation and 3 months post‐treatment initiation) to assess if the LE deficit could be attributed to this choice.

We also report the LE deficit at 30, 45, and 60 years.

All statistical analyses were performed using STATA Version 18.

### 2.4 Ethics

The data were collected for the surveillance of HCV infection, including linkage to treatment data, and death registrations held by the ONS and are covered by Section 251 of the NHS Act 2006 and the Health Service (Control of Patient Information) Regulations 2002 (regulation 3/‘Section 251 Support’).

## 3 Results

Between 2015 and 2022, 92,920 adults diagnosed with HCV were included in the analyses (Appendix 3). The cohort was predominantly male (70.2%), of white ethnicity (78.1%), and 45 years or older (67.5%) (Table 1).

**TABLE 1:** Demographic and clinical characteristics of the treated and untreated cohorts for each period (Note that individuals could contribute to both cohorts in any period if their treatment status changed in that period).

Characteristics				2015–2018								2019–2022				
	Treated				Untreated				Treated				Untreated			
	Total		Deceased		Total		Deceased		Total		Deceased		Total		Deceased	
	*N*	%	*n*	%	*N*	%	*n*	%	*N*	%	*n*	%	*N*	%	*n*	%
All	25,981		753	2.9	72,575		5,059	7.0	57,925		3,819	6.6	61,894		3,663	5.9
Sex																
Female	7,671	29.5	169	2.2	21,765	30.0	1,343	6.2	16,193	28.0	854	5.3	18,713	30.2	1,064	5.7
Male	18,310	70.5	584	3.2	50,810	70.0	37,16	7.3	41,732	72.0	2,965	7.1	43,181	69.8	2,599	6.0
Age at exit																
15–24	31	0.1	0	0.0	342	0.5	17	5.0	204	0.4	4	2.0	583	0.9	10	1.7
25–34	1,017	3.9	19	1.9	3,766	5.2	282	7.5	4,165	7.2	100	2.4	5,230	8.4	142	2.7
35–44	4,811	18.5	86	1.8	16,873	23.2	1,023	6.1	14,822	25.6	597	4.0	17,175	27.7	513	3.0
45–54	7,048	27.1	208	3.0	22,132	30.5	1,509	6.8	17,286	29.8	1047	6.1	18,888	30.5	963	5.1
55–64	7,853	30.2	258	3.3	18,152	25.0	1,216	6.7	13,864	23.9	1,122	8.1	12,692	20.5	859	6.8
65+	5,221	20.1	182	3.5	11,310	15.6	1,012	8.9	7,584	13.1	949	12.5	7,326	11.8	1,176	16.1
Ethnicity																
White	19,786	76.2	610	3.1	57,676	79.5	4,548	7.9	45,375	78.3	3,138	6.9	48,280	78.0	3,040	6.3
Asian	2,545	9.8	41	1.6	5,428	7.5	216	4.0	4,219	7.3	228	5.4	4,153	6.7	266	6.4
Black	1,075	4.1	27	2.5	2,076	2.9	44	2.1	1,759	3.0	126	7.2	1,376	2.2	62	4.5
Other	1,066	4.1	22	2.1	2,990	4.1	120	4.0	1,891	3.3	82	4.3	2,537	4.1	149	5.9
Mixed	280	1.1	4	1.4	511	0.7	4	0.8	591	1.0	30	5.1	367	0.6	3	0.8
Missing	1,229	4.7	49	4.0	3,894	5.4	127	3.3	4,090	7.1	215	5.3	5,181	8.4	143	2.8
Severity																
No fibrosis	8,645	33.3	97	1.1	20,782	28.6	42	0.2	25,817	44.6	1,088	4.2	20,437	33.0	200	1.0
Mild fibrosis	6,581	25.3	86	1.3	12,058	16.6	45	0.4	13,824	23.9	709	5.1	8,305	13.4	44	0.5
Moderate fibrosis	2,719	10.5	42	1.5	4,578	6.3	16	0.3	5,114	8.8	318	6.2	2,770	4.5	44	1.6
Compensated cirrhosis	6,880	26.5	392	5.7	10,276	14.2	67	0.7	10,960	18.9	1,352	12.3	5,271	8.5	115	2.2
Decompensated cirrhosis	621	2.4	119	19.2	1,082	1.5	40	3.7	1,083	1.9	283	26.1	719	1.2	60	8.3
Missing	535	2.1	17	3.2	23,799	32.8	4,849	20.4	1,127	1.9	69	6.1	24,392	39.4	3,200	13.1
Probable route of infection																
Injection drug use	11,312	43.5	329	2.9	29,002	40.0	100	0.3	33,667	58.1	2,246	6.7	26,399	42.7	325	1.2
Blood exposure	2,805	10.8	70	2.5	4,458	6.1	21	0.5	5,518	9.5	324	5.9	3,239	5.2	30	0.9
MSM	765	2.9	10	1.3	1,022	1.4	1	0.1	1,216	2.1	40	3.3	505	0.8	0	0.0
Heterosexual sex	301	1.2	4	1.3	493	0.7	1	0.2	653	1.1	32	4.9	394	0.6	2	0.5
Perinatal	126	0.5	0	0.0	209	0.3	0	0.0	247	0.4	4	1.6	136	0.2	1	0.7
Other	759	2.9	20	2.6	1,128	1.6	4	0.4	1,414	2.4	90	6.4	829	1.3	13	1.6
Missing	9,913	38.2	320	3.2	36,263	50.0	4,932	13.6	15,210	26.3	1,083	7.1	30,392	49.1	3,292	10.8
Treatment region																
London	7,807	30.0	206	2.6	17,079	23.5	879	5.1	13,126	22.7	834	6.4	12,493	20.2	1,046	8.4
North	8,051	31.0	279	3.5	24,811	34.2	1,931	7.8	19,985	34.5	1,458	7.3	22,405	36.2	1,307	5.8
Midlands and East	5,259	20.2	148	2.8	15,327	21.1	1,108	7.2	12,980	22.4	753	5.8	13,568	21.9	636	4.7
South	4,838	18.6	120	2.5	14,247	19.6	1,032	7.2	11,773	20.3	768	6.5	12,146	19.6	581	4.8
Missing	26	0.1	0	0.0	744	1.0	44	5.9	61	0.1	6	9.8	896	1.4	54	6.0
Year of diagnosis																
Pre‐1990	257	1.0	7	2.7	422	0.6	3	0.7	406	0.7	31	7.6	161	0.3	5	3.1
1990–1999	1,709	6.6	48	2.8	3,017	4.2	18	0.6	2,879	5.0	226	7.8	1,285	2.1	21	1.6
2000–2004	2,414	9.3	78	3.2	6,236	8.6	160	2.6	4,507	7.8	380	8.4	3,658	5.9	146	4.0
2005–2009	4,195	16.1	150	3.6	14,754	20.3	698	4.7	7,807	13.5	698	8.9	9,865	15.9	424	4.3
2010–2014	7,866	30.3	278	3.5	22,589	31.1	1,984	8.8	12,831	22.2	982	7.7	12,746	20.6	680	5.3
2015–2019	9,487	36.5	188	2.0	25,557	35.2	2,196	8.6	22,543	38.9	1,366	6.1	20,166	32.6	1,367	6.8
2020–2022	__	__	__	__	__	__	__	__	6,892	11.9	131	1.9	14,013	22.6	1,020	7.3
Missing	53	0.2	4	7.5	__	__	__	__	60	0.1	5	8.3	__	__	__	__
Age at diagnosis																
< 15	173	0.7	2	1.2	556	0.8	3	0.5	308	0.5	7	2.3	390	0.6	3	0.8
15–24	1,510	5.8	18	1.2	4,611	6.4	66	1.4	3,693	6.4	130	3.5	3,557	5.7	53	1.5
25–34	5,561	21.4	86	1.5	18,641	25.7	527	2.8	14,984	25.9	620	4.1	16,128	26.1	339	2.1
35–44	7,325	28.2	145	2.0	21,565	29.7	1,238	5.7	17,761	30.7	10,38	5.8	19,194	31.0	770	4.0
45–54	6,562	25.3	244	3.7	15,974	22.0	1,454	9.1	12,671	21.9	969	7.6	13,016	21.0	903	6.9
55–64	3,636	14.0	178	4.9	7,808	10.8	979	12.5	6,307	10.9	669	10.6	6,263	10.1	663	10.6
65+	1,161	4.5	76	6.5	3,420	4.7	792	23.2	2,141	3.7	381	17.8	3,346	5.4	932	27.9
Missing	53	0.2	4	7.5	__	__	__	__	60	0.1	5	8.3	__	__	__	__

They contributed 322,570 untreated and 200,581 treated person‐years of observation, and 13,294 deaths. Median age of the treated population was 55 years (IQR: 46, 63) in 2015–18 and 51 years (43, 60) in the untreated population. In 2019–22, this was 50 years (42, 59) in the treated population and 49 years (41, 57) in the untreated population.

In 2015–18, there were 5812 deaths resulting in a crude mortality rate of 25.1 (24.4, 25.7) per 1000 person‐years. In 2019–22, there were 7482 deaths resulting in a crude death rate of 25.2 (24.6, 25.7). Between 2015 to 18 and 2019 to 22, there was a reduction in the crude cause‐specific mortality rates for all causes except other cancers, cardiovascular, and respiratory causes (Appendix 4).

In 2015–18, the crude death rate among untreated females was 22.4 (21.2–23.6), 27.5 (26.7–28.4) among untreated males, 16.7 (14.4–19.4) among treated females and 24.3 (22.4–26.4) among treated males. In 2019–22, the crude death rate among untreated females was 25.7 (24.2–27.3), 30.1 (29.0–31.3) among untreated males, 17.9 (16.8–19.2) among treated females and 24.9 (24.1–25.9) among treated males (Table 2).

**TABLE 2:** Characteristics of the study population, and death rates pre‐ and post‐treatment for individuals diagnosed with HCV.

	Treated				Untreated			
	Person‐years	Deaths	2015–18 rate (95% CI)	2019–22 rate (95% CI)	Person‐years	Deaths	2015–18 rate (95% CI)	2019–22 rate (95% CI)
Males								
15–24	618	3	0	5.8 (1.9, 18.1)	2,222	20	10.2 (6.1, 17.3)	7.0 (3.1, 15.6)
25–34	11,218	95	10.1 (6.3, 16.3)	8.2 (6.5, 10.2)	25,439	289	10.9 (9.4, 12.6)	12.3 (10.1, 15.0)
35–44	36,365	507	12.8 (10.0, 16.3)	14.1 (12.9, 15.5)	68,523	1,108	17.5 (16.3, 18.8)	13.9 (12.6, 15.5)
45–54	45,321	1,023	22.0 (19.0, 25.5)	22.7 (21.2, 24.3)	71,047	1,912	27.3 (25.8, 28.9)	26.2 (24.4, 28.2)
55–64	34,318	1,100	29.6 (25.7, 34.1)	32.6 (30.6, 34.8)	38,404	1,620	43.7 (41.0, 46.6)	40.1 (37.2, 43.3)
65–74	12,889	642	49.3 (41.0, 59.3)	49.9 (45.8, 54.3)	12,033	825	61.2 (55.4, 67.6)	76.6 (69.8, 84.1)
75–84	1,930	154	59.1 (38.5, 90.6)	84.4 (71.2, 100.1)	2,788	386	111.7 (96.1, 129.7)	171.0 (149.6, 195.4)
85+	206	25	0	134.9 (91.1, 199.6)	807	155	185.5 (142.5, 237.3)	196.6 (158.5, 241.2)
Total	142,870	3,549	24.3 (22.4, 26.4)	24.9 (24.1, 25.9)	221,267	6,315	27.5 (26.7, 28.4)	30.1 (29.0, 31.3)
Females								
15–24	453	1	0	2.8 (0.4, 19.9)	1,727	7	2.8 (0.9, 8.6)	6.2 (2.3, 16.4)
25–34	5,683	24	2.3 (0.6, 9.1)	4.6 (3.0, 7.0)	14,898	135	9.7 (7.9, 11.8)	7.9 (5.8, 10.7)
35–44	13,312	176	11.5 (7.5, 17.6)	13.5 (11.5, 15.8)	29,977	428	15.4 (13.7, 17.4)	12.6 (10.8, 14.8)
45–54	14,612	232	12.1 (8.6, 17.0)	16.7 (14.6, 19.2)	26,218	560	20.7 (18.6, 23.1)	22.2 (19.6, 25.2)
55–64	14,569	280	21.8 (17.0, 27.8)	18.6 (16.3, 21.2)	16,544	455	27.0 (23.9, 30.4)	28.3 (24.6, 32.5)
65–74	6,698	177	22.5 (15.6, 32.6)	27.3 (23.3, 32.1)	7,368	319	39.8 (34.0, 46.6)	47.2 (40.5, 55.0)
75–84	1,998.	97	44.3 (27.1, 72.3)	49.5 (39.8, 61.5)	3,397	321	80.6 (68.9, 94.3)	112.6 (96.7, 131.1)
85+	382	36	115.3 (48.0, 277.0)	91.4 (62.1, 129.7)	1,169	182	161.9 (129.1, 200.4)	150.6 (122.3, 183.5)
Total	57,711	1,023	16.7 (14.4, 19.4)	17.9 (16.8, 19.2)	101,303	2,407	22.4 (21.2, 23.6)	25.7 (24.2, 27.3)

In 2015–18, the adult LE for untreated females at age 15 was 47.3 years and 55.8 years for treated females at age 15. In a similar time period (2015–17), this was 68.5 for females in the general population according to ONS data. Adult LE at age 15 was 39.4 years and 48.3 years for untreated and treated males, respectively (64.9 from ONS data). In this period, a 15‐year‐old female diagnosed with HCV who received DAA treatment could expect to live 8.5 (95% CI: 5.4–11.5) years longer than one who remained untreated, given their respective prevailing mortality rates. For males, this difference in LE was 8.9 (95% CI: 5.0–11.8) years.

In 2019–22, the adult LE at age 15 was 45.7 years and 52.4 years for untreated and treated females (68.3 between 2019 to 21 from ONS data), and 41.5 years and 46.8 years for untreated and treated males (64.4 from ONS data), respectively. In this period, the adult LE deficit at age 15 for untreated females was 6.7 (95% CI: 3.3–10.0) years and for untreated males was 5.3 (95% CI: 2.9–7.6) years. These differences remained even with changes in the definition of treatment (Appendix 5). These differences also remained significant and appeared more pronounced between 45 to 55 years in 2019–22 when assessed at different ages (Appendix 6 & 7).

**FIGURE 1:**
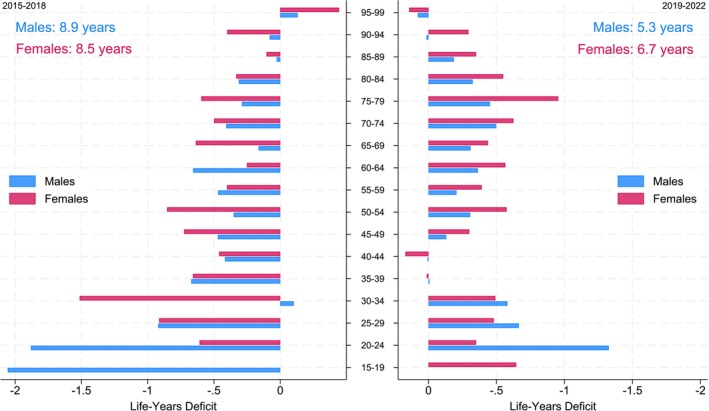
Age contributions to the gross LE deficit for untreated individuals compared to those treated with DAAs in 2015–18 (left) and 2019–22 (right). Note that the axis for 2019–22 has been reversed.

Figure 1 provides insights into age group contributions to the LE deficit for untreated females and males since the introduction of DAAs, with negative values indicating a higher mortality rate among the untreated compared to treated groups. For males, those in younger age groups (15–24) contributed most to the LE deficit in both periods, whereas for females, those aged 30–34 years contributed most to the LE deficit in the 2015–18 period, whereas those aged 75–79 years contributed most in the 2019–22 period.

**FIGURE 2:**
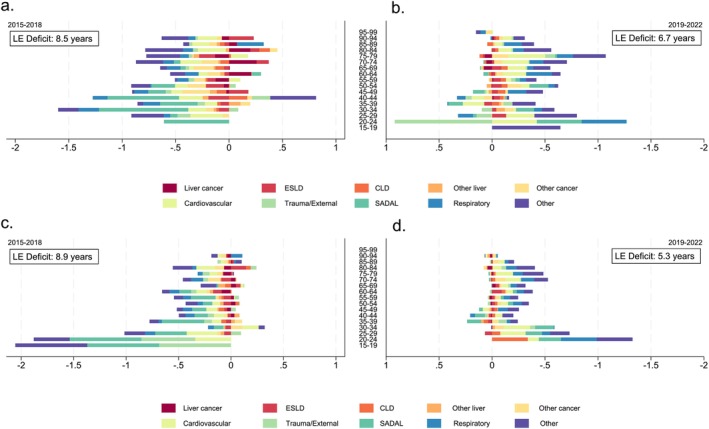
Age and cause of death contributions to the gross LE deficit for untreated individuals compared to those treated with DAAs. Females in 2015–18 (a), Females in 2019–22 (b), Males in 2015–18 (c), and Males in 2019–22 (d).

**FIGURE 3:**
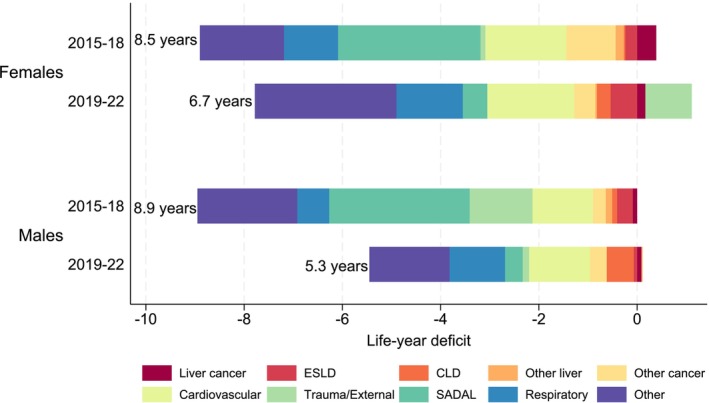
The contribution of cause of death categories to the LE deficits between treated and untreated.

Figure 2 provides insights into the age group and CoD contributions to the LE deficit; CoD contributions aggregated over age are reported in Figure 3. The largest single contributory CoD to the LE deficit in the 2015–18 period was SADAL for both sexes, primarily due to excess mortality in untreated males aged 15–39 years and untreated females aged 20–44 years. In the 2019–22 period, the largest single contributory CoD was cardiovascular causes, closely followed by respiratory causes, for both sexes. Liver‐related causes (HCC, ESLD, CLD, other liver) had a larger contribution to the LE deficit in the 2019–22 period [11.6% (9.0, 14.4) for males, up from 7.1% (5.5, 8.9) and 12.8% (10.2, 15.2) for females up, from 5.3% (3.7, 6.7)]. Crude cause‐specific mortality rates for all liver‐related causes (HCC, ESLD, CLD and other liver causes) fell between period 1 and period 2 in both treated and untreated groups and for both sexes. Crude cause‐specific mortality rates for cardiovascular and respiratory CoD rose for both the treated and untreated groups between 2015 to 18 and 2019 to 22. Additionally, while there was a rise in crude mortality rates due to SADAL in the treated population between the two periods, the untreated population saw a decline in deaths from these causes (although mortality rates from these causes remained higher in the untreated compared to the treated population, with the rates between treated and untreated being extremely close for the 2019–22 period for both males and females) (Table 3, Appendices 8 and 9).

**TABLE 3:** Crude cause‐specific mortality rates (per 1000 person‐years).

Cause of death	Treated				Untreated			
	Females		Males		Females		Males	
	2015–18	2019–22	2015–18	2019–22	2015–18	2019–22	2015–18	2019–22
HCC	2.7 (1.8, 3.9)	1.5 (1.2, 1.9)	3.7 (3.0, 4.6)	2.6 (2.3, 2.9)	1.5 (1.2, 1.9)	1.0 (0.7, 1.4)	2.4 (2.2, 2.7)	2.0 (1.7, 2.3)
ESLD	2.3 (1.4, 3.4)	1.5 (1.1, 1.9)	2.9 (2.2, 3.6)	1.9 (1.7, 2.2)	2.2 (1.9, 2.7)	2.0 (1.6, 2.5)	2.8 (2.5, 3.1)	2.2 (1.9, 2.5)
CLD	0.9 (0.4, 1.7)	0.5 (0.3, 0.7)	1.4 (0.9, 1.9)	0.5 (0.4, 0.7)	0.9 (0.7, 1.2)	0.9 (0.6, 1.3)	1.2 (1.1, 1.4)	0.8 (0.6, 1.0)
Other liver	0.3 (0.1, 0.9)	0.4 (0.2, 0.6)	0.3 (0.1, 0.7)	0.4 (0.3, 0.5)	0.5 (0.4, 0.8)	0.3 (0.1, 0.5)	0.6 (0.4, 0.7)	0.4 (0.2, 0.5)
Other cancer	1.9 (1.1, 2.9)	2.3 (1.9, 2.8)	3.4 (2.7, 4.2)	3.1 (2.8, 3.4)	2.6 (2.2, 3.0)	2.7 (2.2, 3.2)	2.6 (2.3, 2.9)	3.2 (2.8, 3.6)
Cardiovascular	2.6 (1.7, 3.8)	3.1 (2.6, 3.6)	3.3 (2.6, 4.1)	4.2 (3.9, 4.6)	3.2 (2.8, 3.7)	4.8 (4.2, 5.6)	3.6 (3.3, 4.0)	5.5 (5.0, 6.0)
Trauma/External	0.8 (0.3, 1.6)	0.7 (0.5, 1.0)	1.7 (1.2, 2.3)	1.2 (1.0, 1.4)	1.0 (0.8, 1.3)	0.9 (0.7, 1.3)	1.8 (1.6, 2.1)	1.3 (1.1, 1.5)
Alcoholic liver disease	0.3 (0.1, 0.9)	0.5 (0.3, 0.7)	0.4 (0.2, 0.7)	0.4 (0.3, 0.6)	0.6 (0.4, 0.8)	0.3 (0.1, 0.5)	0.7 (0.5, 0.8)	0.4 (0.3, 0.6)
Suicide/Self‐harm	0.3 (0.1, 0.9)	0.2 (0.1, 0.4)	0.7 (0.4, 1.1)	0.4 (0.3, 0.6)	0.4 (0.2, 0.6)	0.2 (0.1, 0.4)	0.6 (0.5, 0.7)	0.6 (0.5, 0.8)
Acute Intoxication	1.6 (0.9, 2.6)	2.7 (2.2, 3.2)	2.7 (2.1, 3.4)	5.0 (4.6, 5.4)	3.6 (3.2, 4.2)	3.2 (2.7, 3.8)	5.4 (5.0, 5.8)	4.9 (4.4, 5.4)
Respiratory	1.1 (0.5, 1.9)	2.2 (1.8, 2.7)	1.2 (0.8, 1.8)	2.7 (2.4, 3.0)	2.2 (1.8, 2.6)	3.9 (3.3, 4.6)	2.0 (1.8, 2.3)	4.0 (3.6, 4.4)
Other	2.1 (1.3, 3.2)	2.3 (1.9, 2.8)	2.5 (1.9, 3.2)	2.4 (2.1, 2.7)	3.6 (3.1, 4.1)	5.3 (4.6, 6.0)	3.7 (3.4, 4.0)	4.7 (4.3, 5.2)
Total	16.7 (14.4, 19.4)	17.9 (16.8, 19.2)	24.3 (22.4, 26.4)	24.9 (24.1, 25.9)	22.4 (21.2, 23.6)	25.7 (24.2, 27.3)	27.5 (26.7, 28.4)	30.1 (29.0, 31.3)
SADAL*	2.2 (1.4, 3.3)	3.4 (2.9, 3.9)	3.8 (3.0, 4.6)	5.9 (5.5, 6.4)	4.6 (4.1, 5.2)	3.7 (3.2, 4.4)	6.7 (6.2, 7.1)	6.0 (5.5, 6.5)
All liver**	6.1 (4.7, 7.8)	3.9 (3.3, 4.5)	8.3 (7.2, 9.6)	5.4 (5.0, 5.8)	5.2 (4.7, 5.9)	4.3 (3.7, 4.9)	7.1 (6.6, 7.5)	5.4 (4.9, 5.9)

* SADAL (Combination of alcoholic liver disease, suicide/self‐harm, and acute intoxication).

** All liver (combination of HCC, ESLD, CLD, and other liver).

## 4 Discussion

Using linked surveillance data (2015–2022), we compared adult LE between DAA‐treated and untreated individuals diagnosed with HCV in England. All‐cause mortality was lower among the treated population, which translated to modest adult LE deficits in the untreated group. In the early DAA period (2015–18), the total adult LE deficit at age 15 was 8.5 (95% CI: 5.4–11.5) years for untreated females and 8.9 (95% CI: 5.0–11.8) years for untreated males. In 2019–22, the deficit narrowed to 6.7 (95% CI: 3.3–10.0) and 5.3 (95% CI: 2.9–7.6) years respectively. In the early DAA period, the deficit was driven by deaths from SADAL, cardiovascular and ‘other’ causes in both sexes (and trauma/external causes in males). In 2019–22, the deficit was primarily driven by ‘other’, cardiovascular and respiratory causes, and an increasing contribution from liver‐related causes. This work adds valuable knowledge to the literature by quantifying the contribution of HCV‐associated mortality to the overall mortality decline in people treated with DAAs. Analyses are disaggregated by sex, thus quantifying any sex differences in the benefits of DAA treatment.

These mortality reductions are consistent with several studies which have assessed the impact of DAAs on mortality with different designs, comparison groups, and outcomes. For example, studies showed reduced incidence and recurrence of HCC and improved survival in those receiving DAAs in Japan [21, 22] and Scotland [23]. Population cohorts in Canada [6] and Australia reported reductions in liver‐ and drug‐related mortality since the introduction of DAAs [24]. Similar findings were reported in France [25] and in veterans [26] and Medicare beneficiaries in the US [27]. However, despite these reductions, mortality in those treated with DAAs remains significantly higher than that in the general population [28].

The observed mortality trends likely reflect historic patterns of HCV incidence. In people diagnosed before the DAA era, there were likely lags in diagnosis and treatment, resulting in long periods between infection and treatment access (a large proportion were diagnosed before 2015). For some of this older cohort, effective treatment arrived too late to have a meaningful impact on mortality, but the impact of treatment is likely to be greater in people who have milder liver disease as a consequence of having been infected for shorter periods of time. We believe the reductions in liver‐related mortality between period 1 and period 2 and the increased contribution of liver‐related causes to the deficit for both sexes in period 2 are early indicators of this. More importantly, as more people receive treatment with DAAs, this may halt the progress of liver deterioration, leading to further reductions in the incidence of both HCC and ESLD among those people who have received treatment and a cure [29]. DAAs will also impact HCV incidence as population viraemia is reduced [30]. It is uncertain how treatment and cure may impact the health and well‐being of people who inject drugs. However, many of those who have acquired their infection more recently have injection drug use as a route of infection [9], and it is this group that is at the highest risk of dying from causes related to drug/alcohol poisoning/overdose and suicide [6, 31, 32]. While clinical cure alone may not fully mitigate these external risks, systematic HCV testing and re‐testing within drug and alcohol services, supported by ODNs, remain essential to engaging this high‐risk population [33, 34].

To the best of our knowledge, this is the first study to decompose the age‐ and cause‐specific contributions to mortality differences between those who have received DAAs and those who are untreated, and only the second to report long‐term benefits of DAAs in a real‐world national dataset. Our results suggest that in the early period (2015–18), treatment was a surrogate for those engaged in care who had more advanced liver disease and less challenging lives, in whom mortality was higher from a range of causes. We know anecdotally that those with evidence of more advanced liver disease were prioritised for treatment and that treatment protocols excluded people with severe comorbidities (people with advanced cancers, severe renal disease and mental illness were not treated). This selection bias likely explains higher liver‐related mortality in the treated group. Lower extrahepatic mortality after treatment could represent extrahepatic benefits of DAA treatment [6, 26] or a selection bias due to systematic exclusion from treatment of individuals with comorbidities.

Notably, SADAL deaths rose among the treated cohort but fell among the untreated in 2019–22, narrowing the LE deficit. We hypothesise that expansion of DAA eligibility meant that a more diverse group of people gained access to treatment in period 2 including people living with poor mental health and those whose drug and alcohol use may have contraindicated them from treatment in the early period combined with the development of in‐reach treatment programmes in prisons and D&A units where shorter duration and/or pan‐genotypic DAA regimens were used to initiate treatment quickly and increase adherence and likelihood of achieving SVR. However, total SADAL deaths remained lower among treated individuals across both periods, which supports findings that treatment might prompt lifestyle changes and adoption of harm reduction strategies [35, 36] or provide protective peer‐support networks [6]. Finally, the universal decline in adult LE during 2019–22 likely represents direct and indirect impacts of the COVID‐19 pandemic on mortality, supported by a doubling of cardiovascular and respiratory mortality across both groups.

The main strength of this study is the large sample size and the use of administrative data, which captures a large percentage of HCV diagnoses, all treatment occurring through the National Health Service (NHS), and all deaths occurring in England. Limitations include the observational nature of the study, which means that we cannot infer causality. There are systematic differences in the data available for treated and untreated populations (e.g., disease severity metrics and liver function are usually assessed at treatment initiation and are often missing for the untreated population), which make more robust causal methods difficult. Secondly, we cannot exclude the possibility of unmeasured confounding, which might exaggerate the benefits of treatment. For example, liver disease severity likely contributes to confounding by indication, and treatment might be confounded by better engagement with healthcare and healthier behaviour in general. Our methods cannot account for confounding due to pandemic effects, making it difficult to attribute mortality differences in this period to treatment alone. Lastly, the use of administrative data means that our analyses were restricted by data availability. A lack of clinical characteristics (e.g., disease‐related markers) for those who remained untreated by the end of the study period meant we could not employ statistical techniques to account for any unmeasured confounding.

Our findings have some important policy implications. First, as more people who inject drugs begin to access treatment, the importance of other strategies like holistic care pathways and harm reduction activities will become more important to sustain the benefits of treatment. This emphasises the continued need for Voluntary, Community and Social Enterprise (VCSE) groups, which may be able to provide this support. Prioritisation of those with more advanced liver disease may have previously delayed positive population impacts, and future studies should evaluate the subsequent move to rapid initiation of DAA treatment for all new cases of HCV to determine the benefits to those with none or minimal liver disease.

This analysis was a first step in quantifying the real‐world differences in mortality associated with DAA treatment; however, mortality is a blunt outcome that likely misses far greater morbidity benefits of treatment. As such, future research will explore associations between DAA treatment and healthcare utilisation.

In conclusion, our findings add to other published evidence that suggests that DAA treatment reduces mortality and also quantifies this reduction using LE deficit. However, reductions in the benefit of treatment in more recent years suggest that treatment programmes will need to evolve to address not just the coordination and delivery of treatment, but also social and behavioural causes of HCV infection to sustain the benefits of treatment.

## Author Contributions

All authors participated in the drafting of the manuscript or critical revision of the manuscript for important intellectual content and provided approval of the final submitted version. Individual contributions are as follows: R.S. prepared and linked all the relevant data. D.E. performed analyses with input from P.T. on classifying causes of death and statistical support from C.S. D.E. drafted a preliminary version of the manuscript. W.R., C.S., and P.T. helped to interpret the findings. W.R., C.S., P.T., and M.D. made revisions for important intellectual content.

## Funding

This work was supported by the National Institute for Health and Care Research (NIHR200911). The research was funded by the National Institute for Health and Care Research Health Protection Research Unit (NIHR HPRU) in Blood Borne and Sexually Transmitted Infections at University College London in partnership with UK HSA. The views expressed are those of the authors and not necessarily those of the NIHR, the Department of Health and Social Care or UKHSA.

## Conflicts of Interest

C.S. has received funding for membership of Advisory Boards, Data Safety and Monitoring Panels and for the preparation of educational materials from Gilead Sciences, ViiV Healthcare, Janssen‐Cilag and M.S.D. P.T. has received honoraria from Gilead Sciences. All other authors declare no competing risks.

## Supporting information


**Appendix 1** Graphical representation of possible trajectories.
Appendix 2 Defining cause of death categories.
Appendix 3 Flow chart of individuals included in the study.
Appendix 4 Crude cause‐specific mortality rates (per 1000 person‐years) and 95% confidence intervals by period.
Appendix 5 Sensitivity analyses varying the treatment status definition to 3 months (i.e., treatment completion) and immediately following treatment initiation.
Appendix 6 Sensitivity analyses looking at LE differences at other ages.
Appendix 7 LE differences at other ages.
Appendix 8 The contribution of cause of death categories to LE differences between treated and untreated groups.
Appendix 9 Crude cause‐specific mortality rates for (a) all causes, (b) liver‐related, (c) cardiovascular, (d) respiratory, (e) SADAL, (f) acute intoxication, (g) suicide, and (h) alcoholic liver disease causes by period and sex.

## Data Availability

Data from this study is not publicly available.
