# Supplementary material for: Trends in Mortality After the Roll‐Out of Directly Acting Antivirals for Hepatitis C in England Using Linked Surveillance Data
Source: J Viral Hepat. 2026 Jul 12;33(8):e70210. doi: 10.1111/jvh.70210 (PMC13358180; doi:10.1111/jvh.70210)
Supplement: Supplementary file 1 — Appendix 1 Graphical representation of possible trajectories. Appendix 2 Defining cause of death categories. Appendix 3 Flow chart of individuals included in the study. Appendix 4 Crude cause‐specific mortality rates (per 1000 person‐years) and 95% confidence intervals by period. Appendix 5 Sensitivity analyses varying the treatment status definition to 3 months (i.e., treatment completion) and immediately following treatment initiation. Appendix 6 Sensitivity analyses looking at LE differences at other ages. Appendix 7 LE differences at other ages. Appendix 8 The contribution of cause of death categories to LE differences between treated and untreated groups. Appendix 9 Crude cause‐specific mortality rates for (a) all causes, (b) liver‐related, (c) cardiovascular, (d) respiratory, (e) SADAL, (f) acute intoxication, (g) suicide, and (h) alcoholic liver disease causes by period and sex. [file JVH-33-0-s001.docx]

**Supplementary material**

Table of Contents

[**Appendix 1: Graphical representation of possible trajectories** 2](#_Toc232792818)

[**Appendix 2: Defining cause of death categories.** 3](#_Toc232792819)

[**Appendix 3: Flow chart of individuals included in the study** 5](#_Toc232792820)

[**Appendix 4: Crude cause-specific mortality rates (per 1,000 person-years) and 95% confidence intervals by period** 6](#_Toc232792821)

[**Appendix 5: Sensitivity analyses varying the treatment status definition to 3 months (i.e., treatment completion) and immediately following treatment initiation** 7](#_Toc232792822)

[**Appendix 6: Sensitivity analyses looking at LE differences at other ages** 8](#_Toc232792823)

[**Appendix 7: LE differences at other ages** 9](#_Toc232792824)

[**Appendix 8: The contribution of cause of death categories to LE differences between treated and untreated groups** 10](#_Toc232792825)

[**Appendix 9:** **Crude cause-specific mortality rates for (a) all causes, (b) liver-related, (c) cardiovascular, (d) respiratory, (e) SADAL, (f) acute intoxication, (g) suicide, and (h) alcoholic liver disease causes by period and sex.** 11](#_Toc232792826)

# **Appendix 1: Graphical representation of possible trajectories**


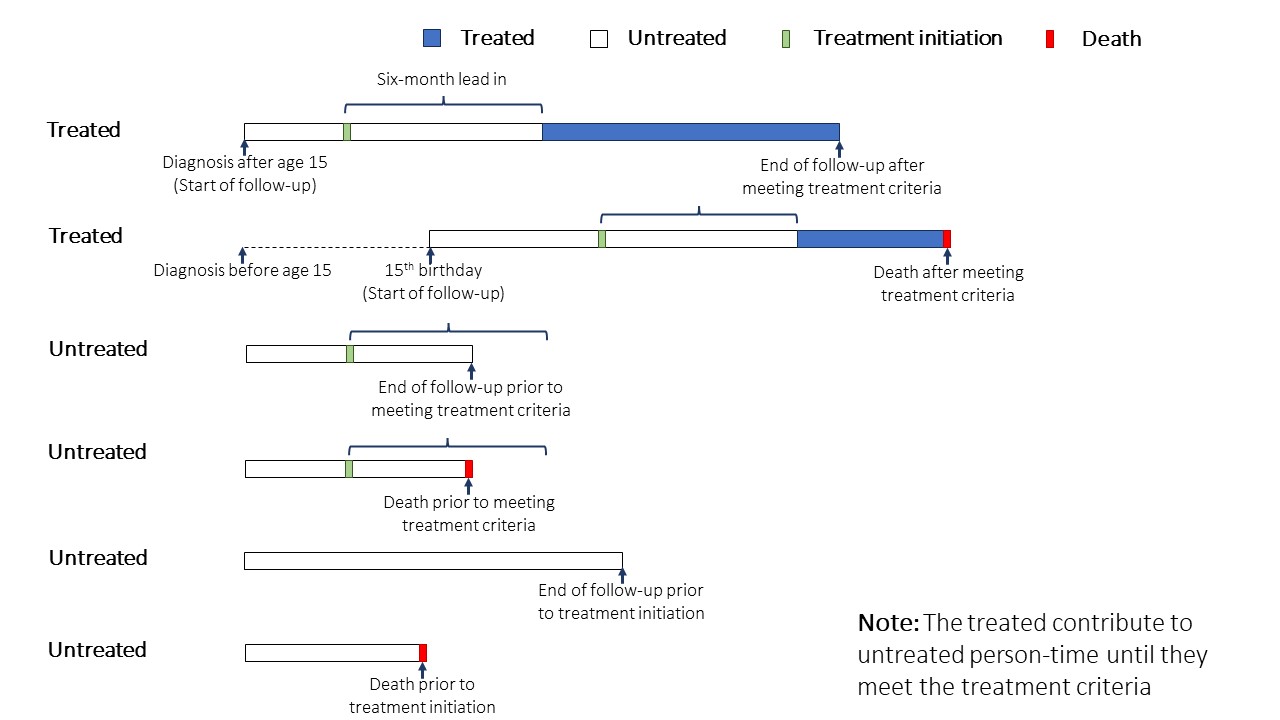


# **Appendix 2: Defining cause of death categories.**

| ICD-10 codes | ICD category | Final category |
| --- | --- | --- |
| C220 C221 C229 C249 D134 D376 | Liver cancer | Liver cancer |
| I850 I983 K704 K720 K721 K729 K767 R18 | End-Stage Liver Disease | End-Stage Liver Disease |
| K732 K738 K739 K740 K746 K750 K758 K759 | Chronic Liver Disease | Chronic Liver Disease |
| K760 K763 K766 K768 K769 | Other Liver | Other Liver |
| B169 B171 B181 B182 B189 B199 | Hepatitis |  |
| C01 C029 C049 C051 C069 C090 C099 C109 C119 C12 C130 C139 C140 C155 C159 C160 C169 C170 C180 C181 C182 C187 C189 C19 C20 C210 C211 C218 C23 C240 C250 C259 C260 C269 C300 C310 C319 C321 C322 C329 C341 C343 C349 C383 C419 C435 C439 C443 C444 C445 C449 C450 C459 C469 C481 C499 C509 C539 C541 C55 C56 C570 C574 C578 C609 C61 C629 C64 C679 C689 C693 C699 C715 C719 C73 C749 C771 C779 C780 C782 C783 C785 C786 C787 C788 C791 C793 C794 C795 C797 C798 C799 C80 C800 C809 C819 C829 C830 C831 C833 C835 C837 C839 C844 C851 C859 C880 C900 C901 C903 C910 C911 C919 C920 C921 C924 C931 C950 C966 C969 D150 D259 D371 D375 D377 D380 D430 D469 D471 D472 D473 D474 D477 D479 D481 D489 | Other cancers | Other cancers |
| I050 I059 I068 I069 I071 I078 I079 I099 I10 I110 I119 I129 I139 I209 I210 I213 I214 I219 I230 I248 I249 I251 I252 I253 I255 I258 I259 I269 I272 I279 I300 I301 I309 I312 I313 I319 I330 I340 I341 I349 I350 I351 I358 I359 I379 I38 I409 I420 I421 I422 I426 I427 I428 I429 I442 I461 I469 I471 I472 I48 I489 I499 I500 I501 I509 I513 I514 I516 I517 I518 I519 I607 I609 I610 I611 I613 I614 I615 I618 I619 I620 I629 I634 I639 I64 I669 I670 I671 I672 I678 I679 I691 I692 I694 I709 I710 I711 I712 I713 I714 I718 I719 1724 I728 I729 I739 I748 I749 I770 I772 I776 I778 I802 I81 I820 I829 I871 I890 I891 | Cardiovascular | Cardiovascular |
| S029 S062 S065 S066 S068 S069 S071 S099 S119 S120 S121 S127 S131 S141 S18 S199 S219 S220 S224 S225 S229 S250 S260 S269 S271 S299 S318 S360 S361 S368 S399 S519 S551 S619 S650 S711 S720 S723 S729 S899 T019 T021 T048 T07 T091 T093 T099 T111 T12 T139 T141 T145 T149 T173 T178 T179 T273 T300 T316 T383 T391 T393 T398 T412 T420 T424 T426 T427 T430 T432 T433 T435 T436 T443 T447 T524 T528 T58 T598 T602 T603 T68 T71 T751 T782 T794 T802 T810 T812 T814 T817 T818 T819 T820 T825 T826 T827 T828 T829 T835 T845 T848 T856 T857 T858 T860 T864 T868 T874 T876 T887 T905 T909 T983 V031 V041 V059 V092 V144 V194 V199 V234 V244 V294 V435 V475 V892 W13 W130 W132 W15 W65 W69 W708 W730 W74 W740 W76 W760 W769 W78 W780 W785 W788 W79 W791 W799 W80 W809 W84 W840 X00 X000 X040 X08 X09 X090 X40 X400 X41 X410 X412 X414 X418 X419 X430 X44 X440 X441 X444 X445 X448 X449 X45 X450 X451 X478 X48 X49 Y09 Y10 Y11 Y14 Y140 Y15 Y150 Y159 Y21 Y218 Y26 Y260 Y280 Y31 Y315 Y33 Y330 Y334 Y339 Y345 Y433 Y434 Y442 Y443 Y445 Y517 Y579 Y608 Y640 Y830 Y831 Y832 Y833 Y834 Y835 Y838 Y839 Y841 Y842 Y844 Y846 Y848 Y86 Y872 Y883 | Trauma and external causes | Trauma and external causes |
| X60 X600 X61 X610 X62 X620 X628 X629 X63 X64 X640 X65 X66 X689 X70 X700 X701 X704 X706 X708 X71 X718 X78 X780 X788 X789 X80 X808 X81 X815 X84 X844 X848 X849 Y20 Y200 Y208 | Suicide or self-harm | SADAL |
| F100 F101 F102 F103 F109 F110 F111 F112 F119 F121 F141 F142 F149 F151 F159 F179 F191 F192 F195 F199 T401 T402 T403 T404 T405 T406 T407 T409 T509 T510 T518 T519 X42 X420 X421 X422 X424 X425 X428 X429 Y12 Y120 Y128 Y129 Y906 | Alcohol or drug toxicity (Acute intoxication) |  |
| K700 K701 K703 K709 | Alcoholic Liver Disease |  |
| A162 A169 A199 A310 B342 B441 B449 B909 J101 J111 J122 J123 J129 J13 J14 J150 J151 J152 J154 J155 J159 J180 J181 J182 J189 J209 J22 J329 J36 J390 J40 J42 J439 J441 J449 J450 J459 J46 J47 J679 J80 J81 J840 J841 J849 J850 J852 J869 J90 J930 J939 J942 J948 J958 J960 J961 J969 J984 J985 J988 J989 U071 U072 U099 U509 | Respiratory | Respiratory |
| AO47 A099 K047 K274 K275 K279 K290 K291 K292 K296 K297 K311 K318 K319 K509 K519 K520 K529 K550 K551 K552 K559 K561 K562 K566 K572 K578 K579 K590 K625 K630 K631 K632 K639 K650 K658 K659 K660 K661 K802 K805 K810 K819 K822 K829 K830 K831 K900 K913 K918 | Gastrointestinal | Other |
| A178 A321 A812 A819 B451 G002 G039 G048 G049 G060 G08 G119 G122 G20 G210 G231 G300 G309 G310 G318 G319 G35 G403 G409 G419 G459 G473 G474 G618 G628 G709 G710 G729 G822 G908 G919 G931 G932 G934 G935 G936 G938 G939 G952 G969 | Nervous system |  |
| B24 B220 | HIV or AIDS |  |
| L022 L024 L028 L029 L031 L039 L088 L089 L120 L409 L539 L893 L899 L97 L984 | Skin disorders |  |
| B029 B589 B962 | Other infectious or parasitic diseases |  |
| D510 D561 D570 D571 D591 D619 D649 D65 D66 D67 D680 D683 D689 D691 D693 D696 D70 D721 D735 D758 D761 D849 D869 D891 | Blood disorders |  |
| E109 E119 E141 E142 E145 E149 E162 E230 E272 E274 E46 E662 E668 E669 E713 E780 E802 E831 E835 E854 E859 E86 E871 E872 E875 E888 E889 | Endocrine, nutritional and metabolic |  |
| F011 F019 F03 F059 F205 F209 F259 F329 F412 F419 F489 F603 F99 | Mental, behavioural, and neurodevelopmental |  |
| M009 M069 M199 M259 M310 M318 M321 M329 M349 M419 M462 M480 M600 M622 M628 M726 M797 M809 M819 M844 M861 M869 | Musculoskeletal |  |
| N009 N019 N028 N049 N052 N059 N10 N111 N119 N12 N133 N139 N179 N180 N184 N185 N189 N19 N26 N280 N289 N300 N309 N321 N390 N40 N47 N498 N508 N735 | Genitourinary |  |
| Q059 Q203 Q211 Q231 Q249 Q279 Q282 Q283 Q612 Q613 Q874 | Congenital malformations |  |
| H602 R99 … | Other causes |  |

# **Appendix 3: Flow chart of individuals included in the study**


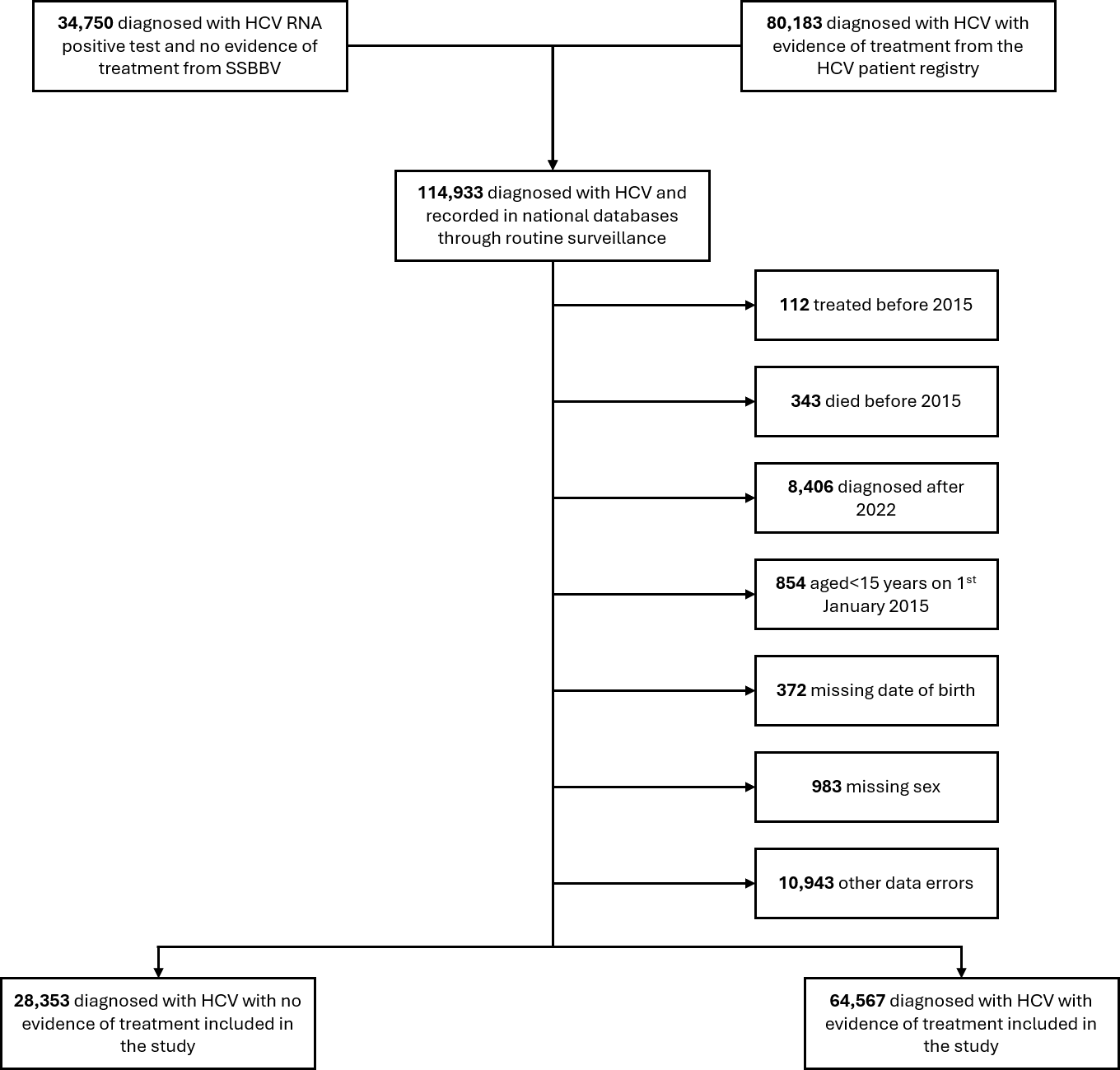


# **Appendix 4: Crude cause-specific mortality rates (per 1,000 person-years) and 95% confidence intervals by period**

|  | Crude cause-specific mortality rates (per 1,000 person-years) | |
| --- | --- | --- |
| Cause of death | **2015-18** | **2019-22** |
| Liver cancer | 2·3 (2·1, 2·5) | 2·0 (1·9, 2·2) |
| ESLD | 2·6 (2·4, 2·8) | 1·9 (1·8, 2·1) |
| CLD | 1·1 (1·1, 1·3) | 0·6 (0·6, 0·7) |
| Other liver | 0·5 (0·4, 0·6) | 0·4 (0·3, 0·4) |
| Other cancer | 2·6 (2·4, 2·8) | 2·9 (2·7, 3·1) |
| Cardiovascular | 3·4 (3·2, 3·7) | 4·5 (4·2, 4·7) |
| Trauma/External | 1·5 (1·4, 1·7) | 1·1 (1·0, 1·2) |
| Alcoholic liver disease | 0·6 (0·5, 0·7) | 0·4 (0·4, 0·5) |
| Suicide/Self-harm | 0·5 (0·4, 0·6) | 0·4 (0·4, 0·5) |
| Acute intoxication | 4·4 (4·2, 4·7) | 4·3 (4·1, 4·6) |
| Respiratory | 1·9 (1·8, 2·1) | 3·1 (2·9, 3·3) |
| Other | 3·4 (3·2, 3·7) | 3·4 (3·2, 3·7) |
| Total | 25·1 (24·4, 25·7) | 25·2 (24·6, 25·7) |

# **Appendix 5: Sensitivity analyses varying the treatment status definition to 3 months (i.e., treatment completion) and immediately following treatment initiation**

| Treatment status assignment | Sex | Period | LE advantage |
| --- | --- | --- | --- |
|  | Females | 2015-18 | 8·5 (5·4, 11·5) |
| 6 months after treatment initiation |  | 2019-22 | 6·7 (3·3, 10·2) |
|  | Males | 2015-18 | 8·9 (5·6, 12·2) |
|  |  | 2019-22 | 5·3 (3·0, 7·6) |
|  | Females | 2015-18 | 8·4 (4·5, 12·3) |
| 3 months after treatment initiation |  | 2019-22 | 5·9 (2·7, 9·1) |
|  | Males | 2015-18 | 8·2 (4·9, 11·5) |
|  |  | 2019-22 | 4·7 (2·3, 7·1) |
|  | Females | 2015-18 | 9·9 (6·3, 12·6) |
| Immediate |  | 2019-22 | 5·1 (1·8, 8·4) |
|  | Males | 2015-18 | 9·4 (6·2, 12·6) |
|  |  | 2019-22 | 3·9 (1·3, 6·5) |

# **Appendix 6: Sensitivity analyses looking at LE differences at other ages**

| Period | 2015-18 | | 2019-22 | |
| --- | --- | --- | --- | --- |
| Sex | Females | Males | Females | Males |
| 15 years | 8·5 (5·4, 11·5) | 8·9 (5·6, 12·2) | 6·7 (3·3, 10·2) | 5·3 (3·0, 7·6) |
| 30 years | 7·0 (4·3, 9·7) | 4·2 (2·7, 5·7) | 5·6 (4·0, 7·2) | 3·4 (2·4, 4·4) |
| 45 years | 4·9 (2·6, 7·2) | 3·8 (2·7, 4·9) | 6·0 (4·6, 7·4) | 3·4 (2·6, 4·1) |
| 60 years | 3·4 (1·0, 5·8) | 3·2 (2·1, 4·3) | 5·9 (4·5, 7·4) | 3·7 (2·9, 4·5) |

# **Appendix 7: LE differences at other ages**


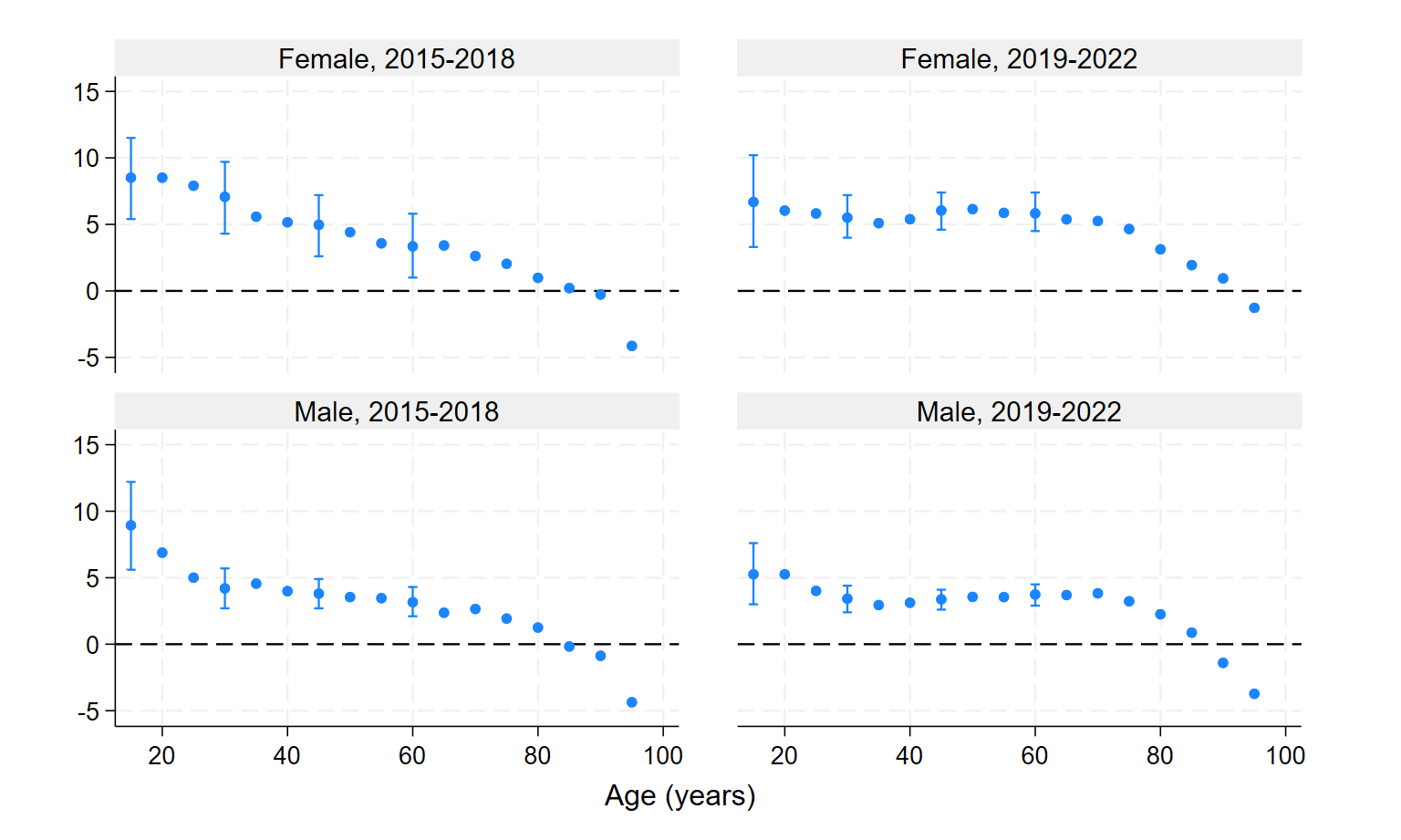


# **Appendix 8: The contribution of cause of death categories to LE differences between treated and untreated groups**

|  | Males |  | Females |  |
| --- | --- | --- | --- | --- |
|  | Years | % | Years | % |
| LE deficit: 2015-18 | 8.9 years |  | 8.5 years |  |
| HCC | 0.09 | 1.0 | -0.39 | __ |
| ESLD | 0.32 | 3.6 | 0.24 | 2.8 |
| CLD | 0.10 | 1.1 | 0.03 | 0.4 |
| Other liver | 0.13 | 1.5 | 0.17 | 2.1 |
| Cancer | 0.26 | 2.9 | 1.00 | 11.8 |
| Cardiovascular | 1.23 | 13.7 | 1.65 | 19.4 |
| Trauma/External | 1.28 | 14.3 | 0.10 | 1.2 |
| SADAL | 2.86 | 32.0 | 2.90 | 34.1 |
| Respiratory | 0.65 | 7.3 | 1.10 | 12.9 |
| Other | 2.03 | 22.7 | 1.71 | 20.1 |
| LE deficit: 2019-22 | 5.3 years |  | 6.7 years |  |
| HCC | -0.09 | __ | -0.17 | __ |
| ESLD | 0.07 | 1.3 | 0.54 | 8.1 |
| CLD | 0.55 | 10.3 | 0.28 | 4.2 |
| Other liver | -0.02 | __ | 0.03 | 0.5 |
| Cancer | 0.34 | 6.4 | 0.43 | 6.4 |
| Cardiovascular | 1.24 | 23.2 | 1.77 | 26.5 |
| Trauma/External | 0.13 | 2.4 | -0.94 | __ |
| SADAL | 0.36 | 6.7 | 0.50 | 7.5 |
| Respiratory | 1.13 | 21.2 | 1.35 | 20.2 |
| Other | 1.63 | 30.6 | 2.88 | 43.2 |

# **Appendix 9:** **Crude cause-specific mortality rates for (a) all causes, (b) liver-related, (c) cardiovascular, (d) respiratory, (e) SADAL, (f) acute intoxication, (g) suicide, and (h) alcoholic liver disease causes by period and sex.**

Note: SADAL (e) is a combination of (f), (g), and (h).
